# Effect and cost-effectiveness of national gastric cancer screening in Japan: a microsimulation modeling study

**DOI:** 10.1186/s12916-020-01729-0

**Published:** 2020-09-14

**Authors:** Hsi-Lan Huang, Chi Yan Leung, Eiko Saito, Kota Katanoda, Chin Hur, Chung Yin Kong, Shuhei Nomura, Kenji Shibuya

**Affiliations:** 1 Department of Global Health Policy, Graduate School of Medicine, The University of Tokyo, Tokyo, Japan; 2 Division of Cancer Statistics Integration, Center for Cancer Control and Information Services, National Cancer Center, Tokyo, Japan; 3 Department of Medicine, Columbia University Irving Medical Center, New York City, USA; 4 Institute for Technology Assessment, Massachusetts General Hospital, Boston, MA USA; 5 Department of Radiology, Harvard Medical School, Boston, MA USA; 6 Department of Health Policy and Management, School of Medicine, Keio University, Tokyo, Japan; 7 Epidemiology and Prevention Group, Center for Public Health Sciences, National Cancer Center, Tokyo, Japan; 8 University Institute for Population Health, King’s College London, London, UK

**Keywords:** Microsimulation, Cost-effectiveness analysis, Gastric cancer, Cancer screening

## Abstract

**Background:**

A national endoscopic screening program for gastric cancer was rolled out in Japan in 2015. We used a microsimulation model to estimate the cost-effectiveness of current screening guidelines and alternative screening strategies in Japan.

**Methods:**

We developed a microsimulation model that simulated a virtual population corresponding to the Japanese population in risk factor profile and life expectancy. We evaluated 15 endoscopic screening scenarios with various starting ages, stopping ages, and screening intervals. The primary outcomes were quality-adjusted life-years (QALYs), costs, and incremental cost-effectiveness ratio. Cost-effective screening strategies were determined using a willingness-to-pay threshold of $50,000 per QALY gained. One-way sensitivity and probabilistic sensitivity analyses were done to explore model uncertainty.

**Results:**

Using the threshold of $50,000 per QALY, a triennial screening program for individuals aged 50 to 75 years was the cost-effective strategy, with an incremental cost-effectiveness ratio of $45,665. Compared with no endoscopic screening, this strategy is predicted to prevent 63% of gastric cancer mortality and confer 27.2 QALYs gained per 1000 individuals over a lifetime period. Current screening guidelines were not on the cost-effectiveness efficient frontier. The results were robust on one-way sensitivity analyses and probabilistic sensitivity analysis.

**Conclusions:**

This modeling study suggests that the endoscopic screening program in Japan would be cost-effective when implemented between age 50 and 75 years, with the screening repeated every 3 years. These findings underscore the need for further evaluation of the current gastric cancer screening recommendations.

## Background

Gastric cancer continues to be a major global health threat, having accounted for 0.8 million deaths and 19.1 million disability-adjusted life-years (DALYs) in 2017 [1]. Results from a recent meta-analysis, which demonstrated a 40% reduction in risk of death from gastric cancer with endoscopic screening [2], shed light on the opportunity to reduce the burden of gastric cancer through effective screening policy. With the third-highest rate of gastric cancer incidence globally [3], Japan introduced a national endoscopic screening program in 2015, offering biennial and triennial endoscopic screening for people older than 50 years [4]. Understanding the trade-offs in lifetime benefits and costs of current screening guidelines, as opposed to alternative screening strategies, at the population level is a vital input into dialogues on cancer control policy. However, with a paucity of empirical evidence and longitudinal data, the lifetime cost-effectiveness of population-wide screening strategies with different screening intervals at various starting and stopping ages remains unclear.

To inform screening policy in a timely fashion, microsimulation decision models can estimate the long-term consequences of a large number of potential policies that are not routinely examined in empirical studies [5, 6]. Here, to identify which strategies might deliver cost-effective care, we developed a microsimulation model which incorporates the best available data to estimate the lifetime cost-effectiveness of various national endoscopic screening scenarios while accounting for individual-level heterogeneity in gastric cancer risk.

## Methods

### Model description

**Fig. 1:**
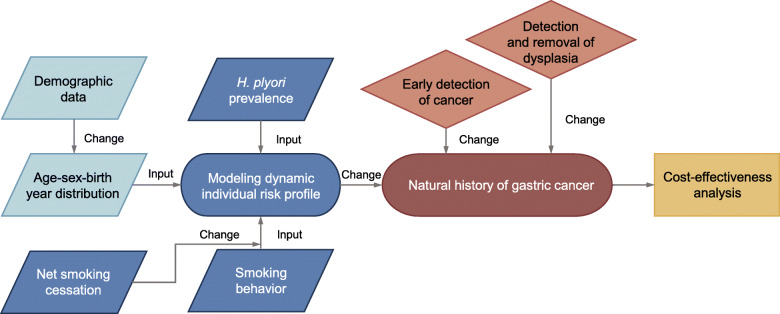
Overview of the modeling logic. Note: Parallelograms represent data input, stadiums represent the process of modeling, diamonds represent intervention, and square indicates model output

We synthesized information from nationally representative data sets on demographics; prevalence of risk exposure; cancer incidence, mortality, and survival; and endoscopic screening (Additional file 1: Table S1) [7–55]. Using these data, we then developed a population-based microsimulation model of gastric cancer and created a virtual population with individual risk profiles and life expectancy which were representative of the population of Japan (Fig. 1).

**Fig. 2:**
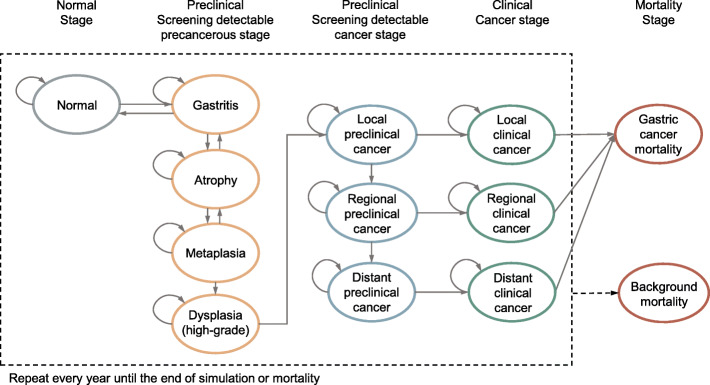
Conceptual framework on the Markov chain of the natural history module. Note: Arrows indicate the transition from one health state to another

The natural history of disease progression was simulated on the basis of Correa’s cascade of gastric carcinogenesis (Fig. 2) [11]. As a simulated individual ages, precancerous lesions (atrophic gastritis, intestinal metaplasia, or dysplasia) may develop. The model focused on non-cardia intestinal-type gastric adenocarcinoma (NCGA), the major histologic type of gastric cancer [12]. The model allows individual risk profiles (smoking behavior and *Helicobacter pylori* infection) to change dynamically and to affect the probability of disease progression over time. Simulated smoking behavior at the individual level depended on respective age, sex, and calendar year and was updated and tracked annually throughout a lifetime [29, 33]. To reflect the secular trend in prevalence across birth cohorts, *H. pylori* infection status was generated according to the birth year when a simulated individual entered the model (Additional file 1: Figure S1) [34]. Preclinical cancer may either become symptomatic, be detected by screening, or progress to a more advanced preclinical cancerous stage. Using long-term survival data from population-based cancer registries, survival time of individuals after cancer diagnosis was simulated by sex, clinical stage, and year after diagnosis [26]. Competing risk of mortality was modeled by respective age, sex, and calendar year [9, 10, 40].

To ground our model in an empirical context, we calibrated and validated the model using population-based cancer registries, which include individual-level, de-identified data on 1.2 million gastric cancer cases diagnosed from 1994 to 2013 [12]. The model was calibrated to the age- and sex-specific incidence and stage distribution of gastric cancer from 2006 to 2008 [12]. We defined the initial search bounds for calibration by conducting a systematic review and explored parameter space systematically by performing 6000 independent searches with 1,000,000 individuals in each search. Of these 6000 resamples, we applied the least-squares method to identify the top 50 best fitted parameter sets and reported model outputs using these 50 parameter sets as uncertainty intervals. To validate the model, we assessed its predictive ability on data not used in the calibration process, namely gastric cancer mortality on vital statistics from 1994 to 2013 [12, 39]. The model was developed using TreeAge Pro 2019 (TreeAge Software Inc., Williamstown, MA). Data was analyzed using STATA 15 (Stata Corp., College Station, TX, USA). The details of the model are described in Additional file 1 and Table S1–S4.

### Scenarios

In addition to current screening recommendations (biennial and triennial endoscopic screening from age 50 with no stopping age), we evaluated 12 strategies with varying starting ages (40, 45, and 50 years), stopping ages (75 and 80 years), and screening intervals (2 and 3 years). Strategies in this report are denoted by starting age-stopping age-screening interval. The baseline scenario was modeled to project the trend of gastric cancer in the absence of a national endoscopic screening policy. After introduction of the endoscopic screening scenarios, the natural history of gastric cancer could then be altered due to the detection of preclinical cancer, or detection or removal of dysplasia, depending on the sensitivity and specificity of the screening tests (Table 1). Individuals with a biopsy result of dysplasia were assumed to be treated by endoscopic submucosal dissection (ESD) and offered yearly surveillance endoscopy for 5 years [37, 69]. Perfect adherence was assumed in all scenarios.

**Table 1:** Key input parameters included in our analysis

Input parameter	Base case analyses	One-way sensitivity analysis range	Probabilistic sensitivity analysis distribution	Source
Endoscopy				
Sensitivity	0.8860000	0.6982–0.976	Beta	[56]
Specificity	0.8510000	0.8430–0.859	Beta	[56]
Complications^a^	0.0000195	Fixed	Beta	[53]
Death	0.0000011	Fixed	Beta	[53]
Endoscopic submucosal dissection				
Complete resection^b^	0.9000000	0.8–1.0	Beta	[54]
Complications^c^	0.0241927	Fixed	Beta	[53]
Death	0.0001599	Fixed	Beta	[53]
Recurrence	0.0140000	Fixed	Beta	[55]
Surgery (gastrectomy)				
Complete resection	1.0000000	Fixed	Fixed	[57, 58]
Complications	0.0615385	Fixed	Beta	[59]
Death	0.0043956	Fixed	Beta	[58, 60]
Recurrence	0.0040000	Fixed	Beta	[55]
Quality of life/utilities				
Endoscopy without complication	− 1 day	Fixed	Fixed	[61]
Endoscopy with complication	− 1 weeks	Fixed	Fixed	[61]
Surgery without complication	− 2 week	Fixed	Fixed	[61]
Surgery with complication	− 1 month	Fixed	Fixed	[61]
Gastric cancer		Fixed	Fixed	
Local	0.773	Fixed	Fixed	[62]
Regional	0.590	Fixed	Fixed	[62]
Distant	0.404	Fixed	Fixed	[62]
Discounting				
Costs	3%	3.5–6%	Fixed	[63]
Quality-adjusted life-years	3%	0–1.5%	Fixed	[63]
Cost (US$)^d^				
Direct				
Endoscopy	127	90–160	Gamma	[64]
ESD	1731	1400–2000	Gamma	[65]
Endoscopy/ESD complication	380	250–450	Gamma	[65]
Endoscopy/ESD complication	852	700–1000	Gamma	[65]
Local cancer, first year	11,110	8000–14,000	Gamma	[64]
Local cancer, subsequent years	1544	Fixed	Gamma	[64]
Regional cancer, first year	20,645	15,000–25,000	Gamma	[64]
Regional cancer, subsequent years	3171	Fixed	Gamma	[64]
Distal cancer, first year	29,610	25,000–35,000	Gamma	[64]
Distal cancer, subsequent years	5655	Fixed	Gamma	[64]
Terminal care year in each stage	51,497	Fixed	Gamma	[64]
Indirect				
Endoscopy, hours	8	Fixed	Fixed	[66]
ESD, hours	56	Fixed	Fixed	[65]
Surgery, hours	136	Fixed	Fixed	[65]
First year of cancer treatment, hours	351	Fixed	Fixed	[67]
Subsequent years of cancer treatment, hours	48	Fixed	Fixed	[67]
Final year of cancer treatment, hours	512	Fixed	Fixed	[67]

*ESD* endoscopic submucosal dissection. ^a^Endoscopic-related screening and diagnostic complications included bleeding and perforation. ^b^Complete resection was defined as resection with tumor-free lateral and vertical margins, without submucosal invasion and lymphovascular invasion. ^c^Complication of endoscopic submucosal dissection included bleeding and perforation. ^d^Costs are presented in 2015 US dollars using an exchange rate of 121. We assumed that the median hourly wage of US$16.75 in 2015 was equivalent to the value of patient time [68]

### Cost-effectiveness analysis

The cost-effectiveness analysis was conducted from a societal perspective in which the model repeatedly simulated 10 million individuals born between 1965 and 1985 in all screening scenarios and followed them from age 20 years until either death or age 100 years. Lifetime screening effectiveness (reduction in gastric cancer mortality, and quality-adjusted life-years [QALYs] gained) and resources (endoscopies and costs) were simulated for each screening strategy. Incremental cost-effectiveness ratios were calculated by dividing the incremental cost by the QALYs gained. QALYs were defined as the product of health utility and time. In this study, one QALY was equivalent to 1 year of perfect health. Incremental cost-effectiveness ratio (ICER) is a metric designed to inform decision-makers of trade-offs when allocating resources to an intervention. In the present study, ICER was calculated by dividing the incremental cost by the QALYs gained [70]. Strategies were ranked by increasing costs. A strategy was dominated if it was more costly but yielded fewer QALYs than its adjacent strategy or had a higher ICER than a more effective strategy. Dominated strategies were excluded, and ICERs were calculated for non-dominated strategies [7]. Costs and QALYs were discounted at an annual rate of 3% [63]. A willingness-to-pay (WTP) threshold of $50,000 US dollars per QALY saved was applied [71]. Costs for gastric cancer screening, diagnosis, and treatment were obtained from the Japanese diagnosis procedure combination-based payment system and published literature and are presented in 2015 US dollars (Table 1) [72]. Table S5 in the Additional file 1 presents a Consolidated Health Economic Evaluation Reporting Standards (CHEERS) checklist.

### Sensitivity analysis

To assess the robustness of results to changes in individual parameters, we performed multiple deterministic sensitivity analyses by varying the sensitivity and specificity of endoscopy and the resection rate of ESD using the reported lower and upper 95% uncertainty bounds (Table 1). The effects of uncertainty surrounding cost inputs of the endoscopic screening examinations, ESD, and cancer treatments at different stages were assessed by varying the costs by ± 20% (Table 1).

In the base case analysis, we applied a discount rate of 3% per annum to both costs and effects, but explored uncertainty as recommended by WHO-choice guidelines using a discount rate of 0% for effects and 6% for costs [63]. We also evaluated differential discounting (1.5% for effects and 3.5% for costs) in sensitivity analysis.

A second-order probabilistic sensitivity analysis was done with a Monte Carlo simulation to investigate the effect of parameter uncertainty on the cost-effectiveness results. The model was run 1000 times, each taking random draws from all inputs with the prespecified uncertainty distributions listed in Table 1.

## Results

### Accuracy of the simulation model

**Fig. 3:**
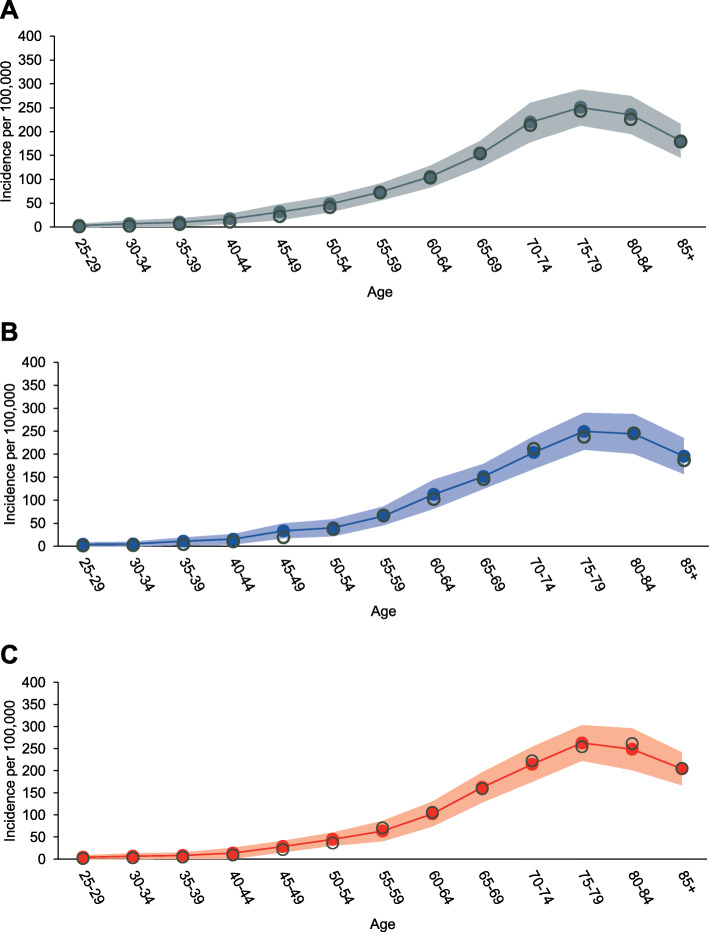
Model predicted gastric cancer incidence. Note: Model calibration shows that the observed and model predicted gastric cancer incidence in **a** 2006, **b** 2007, and **c** 2008 for individuals aged 25 years or above. The observed age-specific gastric cancer incidence is indicated by black hollow dots. The dotted lines indicate the mean predicted age-specific incidence of gastric cancer between 2006 and 2008, and shaded areas indicate uncertainty intervals

Our microsimulation model accurately reproduced the age- and sex-specific incidence rates and stage distributions to the observed trends in population-based cancer registries from 2006 to 2008 (Fig. 3 and Additional file 1: Figure S2–S5). The external-validation analyses also demonstrated long-term coverage estimates of 100% for predicted mortality rates from 1994 to 2013 (Additional file 1: Figure S6).

### Base case analysis

The model estimated that no endoscopic screening resulted in 9.1 gastric cancer mortality, and 47,252 QALYs per 1000 simulated individuals over a lifetime course. Compared with no endoscopic screening, all strategies conferred more QALYs (25.5–32.7 QALYs gained per 1000 individuals) and resulted in reduced simulated rates of gastric cancer mortality (5.7–7.3 events per 1000 individuals). Among 14 screening strategies, lifetime costs were highest in a biennial endoscopic screening program targeting individuals aged 40 to 80 years ($4.2 million per 1000 individuals) and lowest in triennial screening for individuals aged 50 to 75 years ($1.9 million per 1000 individuals). Lifetime number of endoscopies ranged from 9694 per 1000 individuals with the 50-75-3 strategy to 22,358 per 1000 individuals with the 40-80-2 strategy. Detailed base case results are shown in Table 2.

**Table 2:** Estimated lifetime effects and cost-effectiveness of gastric cancer screening strategies

Screening strategies	Lifetime screening outcomes per 1000 individuals					
	GC deaths predicted^**a**^	GC deaths reduction, %^**b**^	Number of endoscopies^**a**^	QALYs gained^**b,c**^	Total costs ($1000)^**c**^	ICER ($ per QALY gained)^**c,d**^
No screening	9.1	0.0	81	0.0	693	–
Current screening guidelines						
50–no stopping age, 3 years	2.3	74.8	14,516	30.1	2247	Dominated
50–no stopping age, 2 years	1.8	80.5	21,379	28.1	3091	Dominated
Alterative screening strategies						
Biennial screening						
Initiation at age 50 years						
50–75, 2 years	3.0	67.3	14,873	25.9	2662	Dominated
50–80, 2 years	2.5	72.3	16,666	27.4	2798	Dominated
Initiation at age 45 years						
45–75, 2 years	2.7	70.3	17,237	26.4	3263	Dominated
45–80, 2 years	2.0	77.9	19,928	28.6	3465	Dominated
Initiation at age 40 years						
40–75, 2 years	2.5	72.7	20,559	25.5	4064	Dominated
40–80, 2 years	2.0	77.6	22,358	26.7	4199	Dominated
Triennial screening						
Initiation at age 50 years						
50–75, 3 years	3.4	63.0	9694	27.2	1934	45,665
50–80, 3 years	2.8	69.1	11,507	29.4	2066	60,731
Initiation at age 45 years						
45–75, 3 years	3.0	67.6	11,907	30.9	2380	Dominated
45–80, 3 years	2.4	73.7	13,660	32.7	2504	130,149
Initiation at age 40 years						
40–75, 3 years	2.8	69.6	14,101	31.1	2909	Dominated
40–80, 3 years	2.4	73.4	15,024	31.5	2975	Dominated

*GC* gastric cancer, *QALYs* quality-adjusted life-years, *ICER* incremental cost-effectiveness ratio. ^a^Gastric cancer deaths and number of endoscopies were not discounted. ^b^Compared with no endoscopic screening. ^c^Discounted at an annual rate of 3%. ^d^Dominated strategies are those either with greater costs and fewer QALYs or with an ICER greater than its adjacent more effective strategy. Dominated strategies are excluded from ICER calculation

**Fig. 4:**
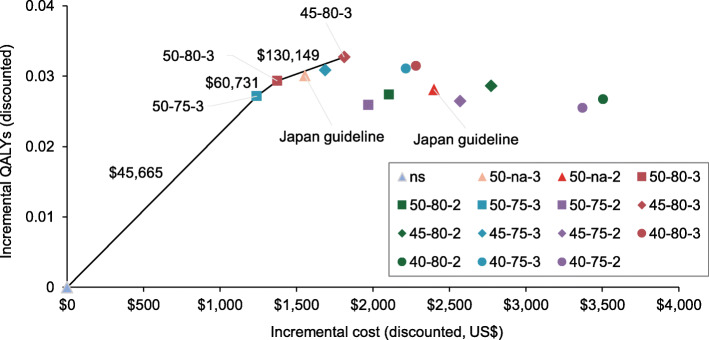
Lifetime cost and quality-adjusted life-year (QALY) of gastric cancer screening strategies compared with no screening. Note: Screening strategies are indicated as starting age-stopping age-screening interval. Costs and QALYs were discounted at an annual rate of 3%. An incremental cost-effectiveness ratio is shown for each strategy on the cost-effectiveness frontier

We computed incremental cost-effectiveness ratios (ICERs) for the non-dominated screening strategies and present the cost-effectiveness frontier in Fig. 4. This frontier was comprised of three triennial screening scenarios: the 50-75-3 strategy, 50-80-3 strategy, and 45-80-3 strategy. Incremental cost-effectiveness ratios were $45,665 for the 50-75-3 strategy compared with no screening, $60,731 for the 50-80-3 strategy compared with the 50-75-3 strategy, and $130,149 for the 45-80-3 strategy compared with the 50-80-3 strategy. Using a WTP threshold of $50,000 per QALY, only triennial screening for individuals aged 50–75 years was cost-effective. This strategy prevented 63% of gastric cancer mortality at an expense of $1.9 million and 9694 endoscopies per 1000 simulated individuals over a lifetime course.

Scenarios simulating the current national endoscopic screening guidelines in Japan, namely biennial and triennial endoscopic screening from age 50 with no stopping age, were not on the efficient frontier. In comparison with no screening, the current biennial screening program would prevent 80.5% of gastric cancer mortality and result in a cost of $3.1 million and 21,379 endoscopies per 1000 simulated individuals. The current triennial screening program would yield a reduction of 74.8% gastric cancer mortality at an expense of $2.2 million and 14,516 endoscopies per 1000 simulated individuals.

### Sensitivity analysis

**Fig. 5:**
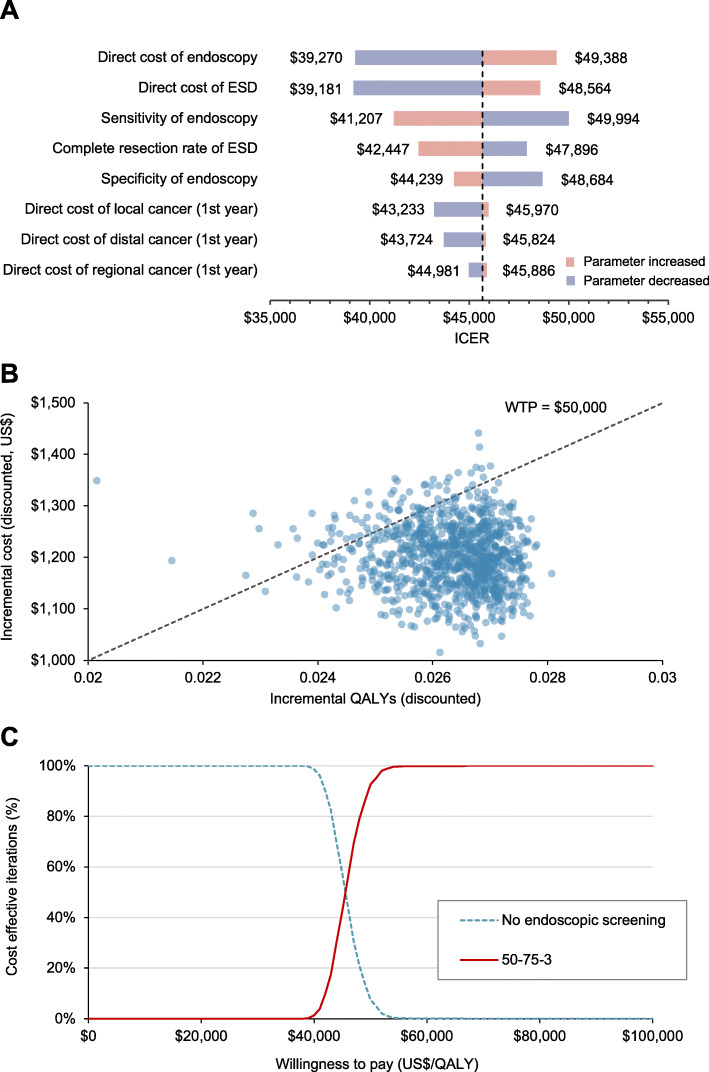
One-way sensitivity analyses and probabilistic sensitivity analysis. Note: ESD, endoscopic submucosal dissection; ICER, incremental cost-effectiveness ratio; QALY, quality-adjusted life-year; WTP, willingness-to-pay threshold. **a** Tornado plot showing ICER estimates for one-way sensitivity analyses. Details of changes to parameter values are given in Table 1. The dotted line indicates ICER from the base case analysis. **b** Result of 1000 bootstraps was generated in the probabilistic sensitivity analysis. Each dot represents the lifetime discounted incremental cost and QALYs of one bootstrap sample. The dotted line indicates willingness-to-pay threshold of US$50000. **c** Cost-effectiveness acceptability curves showing probability that the 50-75-3 strategy is cost-effective across a range of threshold values. The 50-75-3 strategy indicates a triennial screening strategy from 50 to 75 years

The 50-75-3 strategy remained cost-effective under one-way changes to key assumptions. The cost-effectiveness ratio varied between $39,181 and $49,994 per QALY gained (Fig. 5a). The parameters that affect the ICER of the 50-75-3 strategy, from most to least, were the direct cost of endoscopy, direct cost of ESD, sensitivity of endoscopy, complete resection rate of ESD, specificity of endoscopy, first-year medical cost for local cancer, and first-year medical cost for distal cancer and regional cancer. The projected cost-effectiveness results were insensitive to both the use of a 0% discount rate for effects and 6% for the costs ($2736 per QALY gained) and a discount rate of 1.5% for effects and 3.5% for costs ($14,312 per QALY gained).

In our probabilistic sensitivity analysis (Fig. 5b), 100% of the simulations conferred a positive ICER, suggesting that, on a national scale, the 50-75-3 strategy is associated with greater QALYs, although at the expense of a higher cost than no screening. The 50-75-3 strategy was cost-effective in 92.6% of simulations using a WTP threshold of $50,000 per QALY, and in 100% using a WTP threshold of $100,000 per QALY (Fig. 5c).

## Discussion

We developed a well-calibrated and validated microsimulation model to simulate an average-risk population over the lifetime course under 15 unique screening scenarios. Comprehensive modeling showed that the current national endoscopic screening program in Japan is unlikely to be cost-effective. In contrast, a more favorable option would be triennial endoscopic screening of individuals aged 50 to 75 years. This would result in an estimated 27.2 QALYs gained per 1000 individuals and a reduction in the lifetime risk of gastric cancer mortality by 63% with a corresponding ICER of $45,665.

The present study highlights several important policy questions. To date, no recommended age for endoscopic screening cessation has been available. However, with the population aging rapidly, Japan is likely to experience an increased demand for cancer screening despite limited resources. Comprehensive examination of the added value of extending screening practices in older populations is clearly paramount. Our modeling results demonstrate that the benefits of continuing endoscopic screening in people beyond age 75 years do not justify the additional endoscopic screenings performed, owing to diminishing returns. In terms of competing causes of death, adverse events caused by screening, and reduced eligibility for curative surgery in older individuals, determining whether to screen individuals aged over 75 years should be done on an individualized basis, such as with regard to individual risk profile, previous screening history, and the individual’s values and preferences. Further research to explore optimal screening strategies at higher age limits is warranted.

In this analysis, the efficient frontier was dominated by strategies which initiated endoscopic screening at age 50 years; these provided a more favorable option in terms of cost-effectiveness. Our results are in line with the gastric cancer burden in Japan. Data from the population-based cancer registry revealed that the age-specific incidence rate among people aged 40 to 49 years (10.4 per 100,000) was lower than that in people aged 50 to 59 years (65.8 per 100,000) in 2014 [12]. Gastric cancer incidence in these two populations has declined steadily over the past two decades, with reductions in men and women since 1993 of 62.8% and 51.3% at 40 to 49 years compared with 35.8% and 33.4% at 50 to 59 years, respectively (Additional file 1: Figure S7) [12]. For screening effectiveness, age-specific analysis from the Korean national cancer screening program demonstrated that endoscopic screening is related to reduced risk of gastric cancer mortality in the population aged 40 to 74 years [73]. The burden of disease and screening effectiveness, together with the modeling results, indicated that initiating screening at the age of 50 years would be a reasonable option.

To our knowledge, this is the first study to use microsimulation decision modeling to comprehensively project the lifetime cost-effectiveness of national gastric cancer control measures in a high-incidence setting. The strength of this study is that a comprehensive approach was used during formulation of the microsimulation model. This approach incorporated detailed gastric cancer natural history findings and the impact of dynamic individual risk profiles on disease progression by synthesizing the best available data from nationally representative surveys and meta-analyses. This model generates population-level estimates that can hardly be achieved by simpler models, since it also preserves individual-level heterogeneity. In addition, it has been extensively calibrated to the nationally representative observed data. Validation analyses across the period 1994 to 2013 have shown that the secular trends of model predictions are consistent with the observed mortality data. We rigorously modeled 15 clinically relevant scenarios to explore potential lifetime effects across the endoscopic screening spectrum. Further, comprehensive sensitivity analyses were performed, adding robustness to the efficient frontier in this study.

Our study also has several limitations. First, the model assumed full adherence with screening and diagnostic evaluations for all strategies. The current analysis was designed to inform population guidelines; therefore, this assumption allowed the model to predict the maximum achievable benefit of a public health action. Furthermore, to facilitate comparisons, all screening scenarios were based on an identical assumption. Second, because this study focused on current policy and its alternatives, we did not evaluate risk stratification approaches to gastric cancer screening. The combination test of serum pepsinogen and *H. pylori* antibody has been proposed to be a potential tool for predicting gastric cancer development [74–76]. However, the specificity for both single and combination tests of serum pepsinogen and *H. pylori* antibody was shown to be low in one population-based cohort study in Japan [77]. In the future, given that the predictive accuracy of biomarkers could be improved, the risk stratification approach remains a future opportunity which may lead to further enhancement of the cost-effectiveness in a gastric cancer screening context. Lastly, since costs were Japan-based, it is unclear how generalizable our cost efficacy results are to other healthcare systems. However, we have provided both the health benefits and number of endoscopies needed, which are more likely to be generalizable.

## Conclusions

Screening policy could lead to the arrival of a propitious moment in the advancement of gastric cancer control. However, in this microsimulation modeling study, it was estimated that the current national endoscopic screening program in Japan may be less economically attractive than the model-recommended strategy. These findings clearly underpin the need to re-evaluate the current guidelines to develop an efficient policy in Japan.

## Supplementary information

**Additional file 1 : Table S1**. Summary of selected data source for model parameterization. **Table S2**. Key model assumptions. **Table S3**. Net annual smoking cessation rate. **Table S4**. Natural history parameter for calibration and data sources. **Table S5**. CHEERS checklist—Items to include when reporting economic evaluations of health interventions. **Figure S1**. Prevalence of *H. pylori* infection in Japan by birth year from 1908 to 2003. **Figure S2**. Model predicted and observed gastric cancer incidence, both sexes. **Figure S3**. Model predicted and observed gastric cancer incidence, women. **Figure S4**. Model predicted and observed gastric cancer incidence, men. **Figure S5**. Model predicted and observed stage distribution of gastric cancer. **Figure S6**. Predicted gastric cancer mortality. **Figure S7**. Trends in gastric cancer incidence rates by age (ages 40–49 and ages 50–59) and sex, 1993 to 2014.

## Data Availability

Not applicable

## Abbreviations

CHEERS: Consolidated Health Economic Evaluation Reporting Standards
DALYs: Disability-adjusted life-years
ESD: Endoscopic submucosal dissection
ICER: Incremental cost-effectiveness ratio
NCGA: Non-cardia intestinal-type gastric adenocarcinoma
QALYs: Quality-adjusted life-years
WTP: Willingness-to-pay

## References

[1] Etemadi A, Safiri S, Sepanlou SG, Ikuta K, Bisignano C, Shakeri R (2019). The global, regional, and national burden of stomach cancer in 195 countries, 1990–2017: a systematic analysis for the Global Burden of Disease study 2017. Lancet Gastroenterol Hepatol.

[2] Zhang X, Li M, Chen S, Hu J, Guo Q, Liu R (2018). Endoscopic screening in Asian countries is associated with reduced gastric cancer mortality: a meta-analysis and systematic review. Gastroenterology..

[3] Bray F, Ferlay J, Soerjomataram I, Siegel RL, Torre LA, Jemal A (2018). Global cancer statistics 2018: GLOBOCAN estimates of incidence and mortality worldwide for 36 cancers in 185 countries. CA Cancer J Clin.

[4] Hamashima C, Systematic review group and guideline development Group for Gastric Cancer Screening Guidelines (2018). Update version of the Japanese guidelines for gastric cancer screening. Jpn J Clin Oncol.

[5] Van Hees F, Zauber AG, Van Veldhuizen H, Heijnen ML, Penning C, de Koning HJ (2015). The value of models in informing resource allocation in colorectal cancer screening: the case of the Netherlands. Gut..

[6] Owens DK, Whitlock EP, Henderson J, Pignone MP, Krist AH, Bibbins-Domingo K (2016). Use of decision models in the development of evidence-based clinical preventive services recommendations: methods of the US Preventive Services Task Force. Ann Intern Med.

[7] Drummond MF, Sculpher MJ, Claxton K, Stoddart GL, Torrance GW. Methods for the economic evaluation of health care programmes. Third edition. Oxford: Oxford University Press; 2015.

[8] Statistics Bureau. Ministry of Internal Affairs and Communications. Stat Japan. Available from: https://www.e-stat.go.jp/stat-search/database?page=1&toukei=00200524. Accessed 3 Aug 2019.

[9] National Institute of Population and Social Security Research in Japan. Population projection for Japan (2017): 2016 to 2065. Available from: http://www.ipss.go.jp/index-e.asp. Accessed 15 Aug 2019.

[10] Ministry of Health, Labour and Welfare. Life tables: 1999 to 2016 Vital statistics. Available from: http://www.mhlw.go.jp/english/database/db-hw/vs02.html. Accessed 16 July 2019.

[11] Correa P, Piazuelo MB (2012). The gastric precancerous cascade. J Dig Dis.

[12] Hori M, Matsuda T, Shibata A, Katanoda K, Sobue T, Nishimoto H (2015). Cancer incidence and incidence rates in Japan in 2009: a study of 32 population-based cancer registries for the Monitoring of Cancer Incidence in Japan (MCIJ) project. Jpn J Clin Oncol.

[13] Song H, Ekheden IG, Zheng Z, Ericsson J, Nyrén O, Ye W (2015). Incidence of gastric cancer among patients with gastric precancerous lesions: observational cohort study in a low risk Western population. BMJ..

[14] Uemura N, Okamoto S, Yamamoto S, Matsumura N, Yamaguchi S, Yamakido M (2001). Helicobacter pylori infection and the development of gastric cancer. N Engl J Med.

[15] Kato I, Vivas J, Plummer M, Lopez G, Peraza S, Castro D (2004). Environmental factors in Helicobacter pylori-related gastric precancerous lesions in Venezuela. Cancer Epidemiol Prev Biomark.

[16] Adamu MA, Weck MN, Gao L, Brenner H (2010). Incidence of chronic atrophic gastritis: systematic review and meta-analysis of follow-up studies. Eur J Epidemiol.

[17] Russo A, Maconi G, Spinelli P, Di Felice G, Eboli M, Andreola S (2001). Effect of lifestyle, smoking, and diet on development of intestinal metaplasia in H. pylori-positive subjects. Am J Gastroenterol.

[18] González CA, Pardo ML, Ruiz Liso JM, Alonso P, Bonet C, Garcia RM (2010). Gastric cancer occurrence in preneoplastic lesions: a long-term follow-up in a high-risk area in Spain. Int J Cancer.

[19] You WC, Li JY, Blot WJ, Chang YS, Jin ML, Gail MH (1999). Evolution of precancerous lesions in a rural Chinese population at high risk of gastric cancer. Int J Cancer.

[20] Correa P, Haenszel W, Cuello C, Zavala D, Fontham E, Zarama G (1990). Gastric precancerous process in a high risk population: cohort follow-up. Cancer Res.

[21] Plummer M, Vivas J, Lopez G, Bravo JC, Peraza S, Carillo E (2007). Chemoprevention of precancerous gastric lesions with antioxidant vitamin supplementation: a randomized trial in a high-risk population. J Natl Cancer Inst.

[22] den Hoed CM, Holster IL, Capelle LG, de Vries AC, den Hartog B, ter Borg F (2013). Follow-up of premalignant lesions in patients at risk for progression to gastric cancer. Endoscopy..

[23] González CA, Sanz-Anquela JM, Companioni O, Bonet C, Berdasco M, López C (2016). Incomplete type of intestinal metaplasia has the highest risk to progress to gastric cancer: results of the Spanish follow-up multicenter study. J Gastroenterol Hepatol.

[24] Correa P, Fontham ET, Bravo JC, Bravo LE, Ruiz B, Zarama G (2000). Chemoprevention of gastric dysplasia: randomized trial of antioxidant supplements and anti-Helicobacter pylori therapy. J Natl Cancer Inst.

[25] Mansour-Ghanaei F, Joukar F, Soati F, Mansour-Ghanaei A, Atrkar-Roushan Z (2013). Outcome of intestinal metaplasia in gastric biopsy of patients with dyspepsia in Guilan Province, North Iran. Asian Pac J Cancer Prev.

[26] Ito Y, Miyashiro I, Ito H, Hosono S, Chihara D, Nakata-Yamada K (2014). Long-term survival and conditional survival of cancer patients in Japan using population-based cancer registry data. Cancer Sci.

[27] International Agency for Research on Cancer. IARC monographs on the evaluation of carcinogenic risks to humans. Lyon, France: IARC Press; 2011. PMC7681469 1683674

[28] Wong BC, Lam SK, Wong WM, Chen JS, Zheng TT, Feng RE (2004). Helicobacter pylori eradication to prevent gastric cancer in a high-risk region of China: a randomized controlled trial. J Am Med Assoc.

[29] Ministry of Health, Labour and Welfare. Smoking prevalence among Japanese adults (Japan Tobacco Survey). [Japanese]. Available from: http://www.health-net.or.jp/tobacco/product/pd090000.html. Accessed 8 May 2019.

[30] Ministry of Health, Labour and Welfare. Japanese domestic cigarette sales results (Japan Tobacco Survey). [Japanese]. Available from: http://www.health-net.or.jp/tobacco/product/pd070000.html. Accessed 10 May 2019.

[31] Mendez D, Warner KE, Courant PN (1998). Has smoking cessation ceased? Expected trends in the prevalence of smoking in the United States. Am J Epidemiol.

[32] Saito E, Inoue M, Tsugane S, Ito H, Matsuo K, Wakai K (2017). Smoking cessation and subsequent risk of cancer: a pooled analysis of eight population-based cohort studies in Japan. Cancer Epidemiol.

[33] Ministry of Health, Labour and Welfare. National Health and Nutrition Survey. [Japanese]. Available from: http://www.mhlw.go.jp/bunya/kenkou/kenkou_eiyou_chousa.html. Accessed 26 June 2019.

[34] Wang C, Nishiyama T, Kikuchi S, Inoue M, Sawada N, Tsugane S (2017). Changing trends in the prevalence of *H. pylori* infection in Japan (1908–2003): a systematic review and meta-regression analysis of 170,752 individuals. Sci Rep.

[35] Xia HH, Talley NJ (1997). Natural acquisition and spontaneous elimination of Helicobacter pylori infection: clinical implications. Am J Gastroenterol.

[36] Hiroi S, Sugano K, Tanaka S, Kawakami K (2017). Impact of health insurance coverage for Helicobacter pylori gastritis on the trends in eradication therapy in Japan: retrospective observational study and simulation study based on real-world data. BMJ Open.

[37] Kato M, Nishida T, Yamamoto K, Hayashi S, Kitamura S, Yabuta T (2013). Scheduled endoscopic surveillance controls secondary cancer after curative endoscopic resection for early gastric cancer: a multicentre retrospective cohort study by Osaka University ESD study group. Gut..

[38] Kong CY, McMahon PM, Gazelle GS (2009). Calibration of disease simulation model using an engineering approach. Value Health.

[39] Ministry of Health, Labour and Welfare. Vital statistics Japan. Available from: https://ganjoho.jp/reg_stat/index.html. Accessed 8 Mar 2019.

[40] Ministry of Health, Labour and Welfare. Life tables: 1891 to 2000 Vital statistics. Available from: www.stat.go.jp/data/chouki/zuhyou/02-35.xls. Accessed 23 July 2019.

[41] Liu CY, Wu CY, Lin JT, Lee YC, Yen AM, Chen TH (2006). Multistate and multifactorial progression of gastric cancer: results from community-based mass screening for gastric cancer. J Med Screen.

[42] Pittayanon R, Rerknimitr R, Klaikaew N, Sanpavat A, Chaithongrat S, Mahachai V (2017). The risk of gastric cancer in patients with gastric intestinal metaplasia in 5-year follow-up. Aliment Pharmacol Ther.

[43] Coma del Corral MJ, Pardo-Mindan FJ, Razquin S, Ojeda C (1990). Risk of cancer in patients with gastric dysplasia. Follow-up study of 67 patients. Cancer.

[44] Park SY, Jeon SW, Jung MK, Cho CM, Tak WY, Kweon YO (2008). Long-term follow-up study of gastric intraepithelial neoplasias: progression from low-grade dysplasia to invasive carcinoma. Eur J Gastroenterol Hepatol.

[45] Rugge M, Cassaro M, Di Mario F, Leo G, Leandro G, Russo VM (2003). The long term outcome of gastric non-invasive neoplasia. Gut..

[46] Kokkola A, Haapiainen R, Laxen F, Puolakkainen P, Kivilaakso E, Virtamo J (1996). Risk of gastric carcinoma in patients with mucosal dysplasia associated with atrophic gastritis: a follow up study. J Clin Pathol.

[47] Li D, Bautista MC, Jiang SF, Daryani P, Brackett M, Armstrong MA (2016). Risks and predictors of gastric adenocarcinoma in patients with gastric intestinal metaplasia and dysplasia: a population-based study. Am J Gastroenterol.

[48] de Vries AC, van Grieken NC, Looman CW, Casparie MK, de Vries E, Meijer GA (2008). Gastric cancer risk in patients with premalignant gastric lesions: a nationwide cohort study in the Netherlands. Gastroenterology..

[49] Tsukuma H, Oshima A, Narahara H, Morii T (2000). Natural history of early gastric cancer: a non-concurrent, long term, follow up study. Gut..

[50] Iwai T, Yoshida M, Ono H, Kakushima N, Takizawa K, Tanaka M (2017). Natural history of early gastric cancer: a case report and literature review. J Gastr Cancer.

[51] Fujisaki J, Nakajima T, Hirasawa T, Yamamoto Y, Ishiyama A, Tsuchida T (2012). Natural history of gastric cancer—a case followed up for eight years: early to advanced gastric cancer. Clin J Gastroenterol.

[52] Bae JM, Shin SY, Kim EH (2014). Mean sojourn time of preclinical gastric cancer in Korean men: a retrospective observational study. J Prev Med Public Health.

[53] Furuta T, Kato M, Inaba T, Inaba T, Omura N, Katanuma A (2016). 6th report of endoscopic complications: results of the Japan Gastroenterological Endoscopy Society Survey from 2008 to 2012. Gastroenterol Endosc.

[54] Akintoye E, Obaitan I, Muthusamy A, Akanbi O, Olusunmade M, Levine D (2016). Endoscopic submucosal dissection of gastric tumors: a systematic review and meta-analysis. World J Gastrointest Endosc.

[55] Abdelfatah MM, Barakat M, Ahmad D, Ibrahim M, Ahmed Y, Kurdi Y (2019). Long-term outcomes of endoscopic submucosal dissection versus surgery in early gastric cancer: a systematic review and meta-analysis. Eur J Gastroenterol Hepatol.

[56] Hamashima C, Okamoto M, Shabana M, Osaki Y, Kishimoto T (2013). Sensitivity of endoscopic screening for gastric cancer by the incidence method. Int J Cancer.

[57] Meng FS, Zhang ZH, Wang YM, Lu L, Zhu JZ, Ji F (2016). Comparison of endoscopic resection and gastrectomy for the treatment of early gastric cancer: a meta-analysis. Surg Endosc.

[58] Kondo A, de Moura EG, Bernardo WM, Yagi OK, de Moura DT, de Moura ET (2015). Endoscopy vs surgery in the treatment of early gastric cancer: systematic review. World J Gastroenterol.

[59] Li H, Feng LQ, Bian YY, Yang LL, Liu DX, Huo ZB (2019). Comparison of endoscopic submucosal dissection with surgical gastrectomy for early gastric cancer: an updated meta-analysis. World J Gastrointest Oncol.

[60] Hahn KY, Park CH, Lee YK, Chung H, Park JC, Shin SK (2018). Comparative study between endoscopic submucosal dissection and surgery in patients with early gastric cancer. Surg Endosc.

[61] Saumoy M, Schneider Y, Shen N, Kahaleh M, Sharaiha RZ, Shah SC (2018). Cost effectiveness of gastric cancer screening according to race and ethnicity. Gastroenterology..

[62] Lee HJ, Ock M, Kim KP, Jo MW (2018). Estimation of population-based utility weights for gastric cancer-related health states. Patient Preference Adherence.

[63] WHO (2003). Making choices in health: WHO guide to cost-effectiveness analysis.

[64] Saito S, Azumi M, Muneoka Y, Nishino K, Ishikawa T, Sato Y (2018). Cost-effectiveness of combined serum anti-Helicobacter pylori IgG antibody and serum pepsinogen concentrations for screening for gastric cancer risk in Japan. Eur J Health Econ.

[65] Medical News Agency (2019). Diagnosis procedure combination point chart 2019.

[66] Yeh JM, Hur C, Ward Z, Schrag D, Goldie SJ (2016). Gastric adenocarcinoma screening and prevention in the era of new biomarker and endoscopic technologies: a cost-effectiveness analysis. Gut..

[67] Yabroff KR, Davis WW, Lamont EB, Fahey A, Topor M (2007). Patient time costs associated with cancer care. J Natl Cancer Inst.

[68] Ministry of Health, Labour and Welfare. Overview of 2015 basic survey on wage structure. [Japanese]. Available from: https://www.mhlw.go.jp/toukei/itiran/roudou/chingin/kouzou/z2015/. Accessed 12 Sept 2019.

[69] Ono H, Yao K, Fujishiro M, Oda I, Nimura S, Yahagi N (2016). Guidelines for endoscopic submucosal dissection and endoscopic mucosal resection for early gastric cancer. Dig Endosc.

[70] Sanders GD, Maciejewski ML, Basu A (2019). Overview of cost-effectiveness analysis. JAMA..

[71] Shiroiwa T, Sung YK, Fukuda T, Lang HC, Bae SC, Tsutani K (2010). International survey on willingness-to-pay (WTP) for one additional QALY gained: what is the threshold of cost effectiveness?. Health Econ.

[72] The World Bank. GDP deflator: linked series (base year varies by country). Available from: https://data.worldbank.org/indicator/NY.GDP.DEFL.ZS.AD. Accessed 17 Sept 2019.

[73] Jun JK, Choi KS, Lee HY, Suh M, Park B, Song SH (2017). Effectiveness of the Korean National Cancer Screening Program in reducing gastric cancer mortality. Gastroenterology..

[74] Miki K (2011). Gastric cancer screening by combined assay for serum anti-Helicobacter pylori IgG antibody and serum pepsinogen levels—“ABC method”. Proc Japan Acad Series B.

[75] Terasawa T, Nishida H, Kato K, Miyashiro I, Yoshikawa T, Takaku R, et al. Prediction of gastric cancer development by serum pepsinogen test and Helicobacter pylori seropositivity in Eastern Asians: a systematic review and meta-analysis. PLoS One. 2014;9:e109783. 10.1371/journal.pone.0109783 PMC4196955 25314140

[76] Ikeda F, Shikata K, Hata J, Fukuhara M, Hirakawa Y, Ohara T, et al. Combination of Helicobacter pylori antibody and serum pepsinogen as a good predictive tool of gastric cancer incidence: 20-year prospective data from the Hisayama study. J Epidemiol. 2016;26:629­–36. 10.2188/jea.JE20150258 PMC5121431 27265836

[77] Hamashima C, Sasazuki S, Inoue M, Tsugane S, JPHC study group (2017). Receiver operating characteristic analysis of prediction for gastric cancer development using serum pepsinogen and Helicobacter pylori antibody tests. BMC Cancer.

