# Supplementary material for: Effect and cost-effectiveness of national gastric cancer screening in Japan: a microsimulation modeling study
Source: BMC Med. 2020 Sep 14;18:257. doi: 10.1186/s12916-020-01729-0 (PMC7489209; doi:10.1186/s12916-020-01729-0)
Supplement: Supplementary file 1 — Additional file 1 : Table S1. Summary of selected data source for model parameterization. Table S2. Key model assumptions. Table S3. Net annual smoking cessation rate. Table S4. Natural history parameter for calibration and data sources. Table S5. CHEERS checklist—Items to include when reporting economic evaluations of health interventions. Figure S1. Prevalence of H. pylori infection in Japan by birth year from 1908 to 2003. Figure S2. Model predicted and observed gastric cancer incidence, both sexes. Figure S3. Model predicted and observed gastric cancer incidence, women. Figure S4. Model predicted and observed gastric cancer incidence, men. Figure S5. Model predicted and observed stage distribution of gastric cancer. Figure S6. Predicted gastric cancer mortality. Figure S7. Trends in gastric cancer incidence rates by age (ages 40–49 and ages 50–59) and sex, 1993 to 2014. [file 12916_2020_1729_MOESM1_ESM.docx]

**Effect and cost-effectiveness of national gastric cancer screening in Japan: a microsimulation modeling study**

**Supplementary Appendix**

*Supplementary tables*

**Table S1.** Summary of selected data source for model parameterization

**Table S2.** Key model assumptions

**Table S3.** Net annual smoking cessation rate

**Table S4.** Natural history parameter for calibration and data sources

**Table S5.** CHEERS checklist—Items to include when reporting economic evaluations of health interventions

*Supplementary figures*

**Figure S1.** Prevalence of *H. pylori* infection in Japan by birth year from 1908 to 2003

**Figure S2.** Model predicted and observed gastric cancer incidence, both sexes

**Figure S3.** Model predicted and observed gastric cancer incidence, women

**Figure S4.** Model predicted and observed gastric cancer incidence, men

**Figure S5.** Model predicted and observed stage distribution of gastric cancer

**Figure S6.** Predicted gastric cancer mortality

**Figure S7.** Trends in gastric cancer incidence rates by age (ages 40–49 and ages 50–59) and sex, 1993 to 2014

**Model overview**

We developed the Health Simulation Model of Gastric Cancer (HSIM-GC) as a discrete time dynamic stochastic microsimulation model of the natural history and screening of gastric cancer. To inform national policy on gastric cancer screening, the model was adapted to project lifetime costs and health effects under 15 endoscopic screening scenarios in the Japanese context.

Microsimulation (also called 1st-order Monte Carlo simulation or individual-level simulation) is a simulation approach that simulates life histories at an individual level based on biological, demographic, and epidemiologic data [7]. Also, microsimulation tracks discrete events along the path of the individual’s life. This offers a model with great flexibility as individuals’ disease progression could depend on past events [7].

We integrated information from nationally representative data sets to formulate the model (Table S1). The simulations of each individual begin from age 20 years and continue in discrete annual steps until either death or age 100 years. Our model consists of three interactive modules: 1) a demographic module, 2) a natural history module, and 3) a screening module. Each of the modules is detailed in the following sections. The key model assumptions are listed in Table S2. We performed extensive calibration and validation of the model against data on population-based cancer registries and vital statistics. The model was developed in TreeAge Pro 2019 (TreeAge Software Inc, Williamstown, MA).

**Table S1. Summary of selected data source for model parameterization**

| **Parameter** | **Year** | **Data source** | **eReference** |
| --- | --- | --- | --- |
| Demographic module |  |  |  |
| Population estimates by sex | 1920–2015 | Statistics Bureau, Ministry of Internal Affairs and Communications of Japan | [8] |
|  | 2016–2065 | National Institute of Population and Social Security Research | [9] |
| Background mortality by age and sex | 1920–2016 | Vital Statistics in Japan, Ministry of Health, Labour and Welfare, Japan | [10,40] |
|  | 2017–2065 | National Institute of Population and Social Security Research | [9] |
| Natural history module |  |  |  |
| Long-term cancer survival from population-based cancer registry data by stage and sex | 2002–2006 | Center for Cancer Control and Information Services, National Cancer Center, Japan | [26] |
| Smoking prevalence by age and sex | 1965–2017 | Japan Tobacco Incorporated | [29] |
| Number of cigarettes smoked per day by age and sex | 1995–2015 | National Health and Nutrition Survey, Ministry of Health, Labour and Welfare of Japan | [33] |
| Prevalence of *H. pylori* infection by birth year | 1908–2003 | Published meta-analysis | [34] |
| Transition probabilities of disease progression | – | Systematic review ^a^ (see Table S3) | [13,18–25,41–52] |
| Screening module |  |  |  |
| Complications of screening endoscopy | 2008–2012 | National survey of the Japan Gastroenterological Endoscopy Society | [53] |
| Treatment outcomes of therapeutic endoscopy | – | Published meta-analysis | [54,55] |
| Complications of therapeutic endoscopy | 2008–2012 | National survey of the Japan Gastroenterological Endoscopy Society | [53] |
| Model calibration |  |  |  |
| Cancer incidence by age and sex | 2006–2008 | Center for Cancer Control and Information Services, National Cancer Center, Japan | [12] |
| Stage distribution by age and sex | 2006–2008 | Center for Cancer Control and Information Services, National Cancer Center, Japan | [12] |
| Model validation |  |  |  |
| Cancer mortality | 1994–2013 | Center for Cancer Control and Information Services, National Cancer Center, Japan | [39] |

Note: ^a^ Systematic review conducted by the author, *H. pylori*: *Helicobacter pylori*.

**Table S2. Key model assumptions**

| **Descriptor** | **Assumption** |
| --- | --- |
| Demographic | Birth cohort: 1965 to 1985 |
|  | Individuals entered the model at the age of 20 years with no history of cancer. |
|  | Individuals who survived cancer mortality or background mortality were assumed to have died at the age of 100 years. |
|  | Background mortality was modelled by respective age, sex, and calendar year. |
|  | Background mortality was independent of gastric cancer mortality. |
|  | Population structure was modelled to the relative size of each birth cohort. |
|  | Emigration and immigration were not modelled. |
| Natural history | Disease progression was simulated on the basis of Correa’s cascade of gastric carcinogenesis. |
|  | Health states included: normal gastric mucosa, gastritis, atrophic gastritis, intestinal metaplasia, high-grade dysplasia, local preclinical cancer, regional preclinical cancer, distant preclinical cancer, local clinical cancer, regional clinical cancer, distant clinical cancer, and mortality |
|  | Gastritis, atrophic gastritis, and intestinal metaplasia could regress to a less advanced lesion. |
|  | Gastric cancer can only progress from high-grade dysplasia. |
|  | Risk factors classified as a human carcinogen (Group 1) for gastric cancer by the International Agency for Research on Cancer (IARC) were incorporated: smoking behavior and *H. pylori* infection. |
|  | *H. pylori* infection rate can vary by birth cohort. Infection status was assumed to maintain unless successfully treated with antibiotics. |
|  | Smoking behaviors of simulated smokers can differ on the basis of age- and sex-specific population distribution and by calendar year. |
|  | Gastric cancer can be detected either by symptoms or screening. |
|  | Individuals diagnosed with gastric cancer were assumed to adhere to stage-specific treatment regimens. |
|  | Individual’s probability of dying from gastric cancer was simulated according to sex, clinical stage, and years after clinical diagnosis, using the long-term survival data from population-based cancer registries. |
| Screening | Perfect adherence was assumed in all scenarios. |
|  | Endoscopy sensitivity, specificity, and complication rates were assumed to be the same throughout an individual’s life. |
|  | Test sensitivity and specificity in a given round were assumed to be independent of the subsequent screening round. |
|  | Individuals with a biopsy result of dysplasia were assumed to be treated by endoscopic submucosal dissection and offered yearly surveillance endoscopy for five years. |

Note: *H. pylori*: *Helicobacter pylori*

**Demographic module**

In this module, we integrated data on population estimates and life tables from the Statistics Bureau, Ministry of Internal Affairs and Communications [8]; the National Institute of Population and Social Security Research [9]; and the Ministry of Health, Labour and Welfare [10]. Using these data, we developed a demographic module to simulate the population structure and distribution of deaths. To model the age structure of a population that changes over time, successive new individuals born in 1900 to 2015 entered the model at age of 20 years. Each individual in this age-structured population was simulated in parallel. For each individual in the absence of gastric cancer, a year of background mortality (deaths from causes other than gastric cancer) was modelled. Our model allows background mortality to be simulated by respective age, sex and calendar year, in which individuals have risks of death from other causes at any age. In this module, we did not explicitly model population migration.

**Natural history module**

The natural history of disease progression was simulated on the basis of Correa’s cascade of gastric carcinogenesis [11]. The model focused on non-cardia intestinal-type gastric adenocarcinoma (NCGA), the major histologic type of gastric cancer [12]. Unlike other histologic subtypes, the precancerous development process of NCGA is well-defined, from normal to non-atrophic gastritis, then atrophic gastritis, intestinal metaplasia, dysplasia, and finally adenocarcinoma [11]. Atrophic gastritis, metaplasia, and dysplasia confer a high gastric cancer risk and are considered to be precancerous conditions [11,13,14]. In addition, epidemiological evidence has well described the influence of risk factors (cigarette smoking and *Helicobacter pylori* infection) on the carcinogenesis process [15–17].

The progression of gastric cancer was divided into 11 discrete health states: normal gastric mucosa, gastritis, atrophic gastritis, intestinal metaplasia, dysplasia, local preclinical cancer, regional preclinical cancer, distant preclinical cancer, local clinical cancer, regional clinical cancer, and distant clinical cancer. There were two death states: background mortality and gastric cancer mortality. Each year, individuals in the simulation can move from one health state to another or stay in the same health state according to the transition probability of a given health state. Individual risk profile (described below) affects the transition probabilities and is updated and tracked each year throughout the individual’s lifetime, thereby giving dynamic changes in transition probabilities over time. The model assumed that a precancerous lesion, except for dysplasia, could regress into a less advanced lesion [18–25]. As the simulated individual ages, precancerous lesions (atrophic gastritis, intestinal metaplasia, or dysplasia) may develop. Preclinical cancer may become symptomatic, or be detected by screening, or progress to a more advanced preclinical cancerous stage. The model simulated the effects of cancer treatment and survival after cancer diagnosis. After being diagnosed with gastric cancer, individuals in the model have the probability of dying from background cause of death plus an additional probability of dying from gastric cancer in each cycle. Using the long-term survival data from population-based cancer registries, an individual’s probability of dying from gastric cancer was simulated according to sex, clinical stage, and years after clinical diagnosis [26].

**Individual risk profile**

In this study, we only modelled risk factors which have been classified as a human carcinogen (Group 1) for gastric cancer by the International Agency for Research on Cancer (IARC), namely smoking behavior and *H. pylori* infection [15–17,27]. Our model allows individual risk profile to change dynamically and to affect the probability of disease progression overtime, therefore giving dynamic change to the model. Based on epidemiological evidence, we assumed that a smoking history increases the transition probability for progression to metaplasia and to dysplasia [15,17], while *H. pylori* infection increases the transition probability from non-atrophic gastritis to atrophic gastritis [16]. In the model, we based the impact of *H. pylori* infection and the effect of eradication therapy on the results of a randomized controlled trial conducted by Wong et al [28].

Individual smoking behavior was simulated on the basis of the respective age, sex, and calendar year, and was updated and tracked every year throughout life. We derived smoking prevalence by age and sex for 1965–2017 from an annual survey by Japan Tobacco Incorporated (JT) [29], which categorized the subjects surveyed into smokers and non-smokers [29]. Smoking prevalence was projected backward to 1920 by logistic regression using Japanese domestic cigarette sales results reported by JT as a covariate [30]. Using these data, we generated the smoking status of synthetic individuals at age 20 years by sex and calendar year. To reflect the impact of successive tobacco tax increases (2006 and 2010) on net smoking cessation probability in Japan, we calculated the net smoking cessation probabilities by sex in three time periods (before 2007, 2007–2010, and after 2010) using the method proposed by Mendez et al. (Table S3) [31]. Smoking cessation probabilities by sex and time horizon were used to determine whether a simulated individual quit smoking and the year of smoking cessation. Our model also tracked the history of smoking cessation and incorporated its impact on disease progression [32]. According to a pooled estimate from eight population-based cohort studies in Japan, the risk of disease progression among former smokers remains at the same level for 20 years after cessation and declines to never-smoker level for those quitting more than 20 years [32]. Our model simulated the dose-response of smoking and cancer development [15], in which smokers were stratified into two categories: less or more than 10 cigarettes per day. Once a simulated person initiates cigarette smoking, the number of cigarettes smoked per day was generated annually according to respective age, sex, and calendar year until smoking cessation or death. Age- and sex-specific data on number of cigarettes smoked per day from 1995 to 2015 was obtained from the annual National Health and Nutrition Survey [33].

To reflect the secular trend in prevalence across birth cohorts, *H. pylori* infection status (infected or not) was generated according to birth year when simulated individuals entered the model (Figure S1) [34], and assumed to remain constant over the lifetime unless successfully treated with antibiotics [35]. The incorporation of long-term birth cohort-specific data allowed us to account for the trend in *H. pylori* prevalence owing to the effect of eradication therapy, which was covered by the national health insurance scheme beginning 2000 [36].

**Table S3. Net annual smoking cessation rate**

| **Time period** | **Net smoking cessation rate (Men)** | **Net smoking cessation rate (Women)** |
| --- | --- | --- |
| 1920–2007 | 0.0469668 | 0.0551262 |
| 2007–2010 | 0.0528438 | 0.0446247 |
| > 2010 | 0.0722723 | 0.0637806 |

**Figure S1. Prevalence of *H. pylori* infection in Japan by birth year from 1908 to 2003**

|  |
| --- |

Note: The line indicates prevalence of *H. pylori* infection in Japan, and shaded area, indicates 95% confidence intervals for the predicted prevalence. The graph was reproduced based on the data from published meta-analysis [34].

**Natural history parameters**

To define the initial search bounds for model calibration, we searched PubMed, Embase, and the Cochrane library for potential studies from inception to August 2019 reporting plausible ranges of all transition probabilities on disease progression. We applied no language restrictions. Articles were searched using the keywords and Mesh terms “gastritis”, “atrophic”, “metaplasia”, “dysplasia”, “precancerous”, and “gastric cancer”, along with other relevant terms. In addition, we searched reference lists of identified articles. Two reviewers (HLH and CYL) independently screened the title and abstract of potentially eligible articles for inclusion. After initial literature searching and exclusion of duplicated articles, we identified 11394 articles. We rejected a further 11353 articles after reviewing titles and abstract, because they were not relevant to the scope of our study. For the 41 articles under full-text review, 21 were excluded due to inappropriate outcomes and missing data, leaving 20 articles for model parameterization on transition probability. Table S4 lists the plausible range of parameters identified from the included studies.

**Table S4. Natural history parameter for calibration and data sources**

| **Parameter** | **Plausible range** | **eReference** |
| --- | --- | --- |
| Transition probability | Annual probability |  |
| Normal to gastritis | 0.0360531 | [18] |
| Gastritis to atrophic gastritis | 0.0085721–0.1471742 | [18–21] |
| Atrophic gastritis to metaplasia | 0.0003549–0.2423244 | [18–24,41] |
| Metaplasia to dysplasia | 0.0040131–0.1235880 | [19–25,42] |
| Dysplasia to local preclinical cancer | 0.0023263–0.0675237 | [13,19,24,43–48] |
| Gastritis to normal | 0.0043724 | [18] |
| Atrophic gastritis to gastritis | 0.0229958–0.1631556 | [18–24] |
| Metaplasia to atrophic gastritis | 0.0053968–0.0878949 | [18–25] |
| Dysplasia to metaplasia | 0.0257995–0.2831673 | [19–24] |
| Preclinical local cancer to preclinical regional cancer | 0.1229316–0.2606017 | [49–51] |
| Preclinical regional cancer to preclinical distant cancer | 0.3934693 | [50] |
| Preclinical cancer to clinical cancer | 0.2346176–0.5506710 | [52] |
| Risk factors influence transition probability | Relative risk |  |
| *H. pylori* |  |  |
| Gastritis to atrophic gastritis | 3.1–8.3 | [16] |
| Smoking |  |  |
| Atrophy to metaplasia |  |  |
| Smokers (<=10 cigarettes per day) | 1.0–1.3 | [15] |
| Smokers (>10 cigarettes per day) | 1.1–1.7 | [15] |
| Former smokers (quit smoking <=20 years) | 1.0–1.5 | [15] |
| Metaplasia to dysplasia |  |  |
| Smokers (<=10 cigarettes per day) | 1.0–2.8 | [15] |
| Smokers (>10 cigarettes per day) | 2.1–6.0 | [15] |
| Former smokers (quit smoking <=20 years) | 1.0–3.1 | [15] |

Note: *H. pylori*: *Helicobacter pylori*.

**Screening module**

The development of gastric cancer could be interrupted by screening scenarios. With endoscopic screening, dysplasia could be found and removed; and pre-clinical cancer could be diagnosed and treated at an earlier stage. Individuals with a biopsy result of dysplasia were assumed to be treated with endoscopic submucosal dissection [19] and offered yearly surveillance endoscopy for 5 years [37]. However, dysplasia and cancer may be missed due to the imperfect test sensitivity and interval cancers could occur between screening rounds. We assumed that test sensitivity and specificity in a given round were independent of the subsequent screening round. To assess the potential effects of variations in screening test sensitivity and specificity on cost effectiveness results, we performed multiple deterministic sensitivity analyses and probabilistic sensitivity analysis.

In addition to current screening recommendations (biennial and triennial endoscopic screening from age 50 with no stopping age), we evaluated 12 strategies with varying starting ages (40, 45, and 50 years), stopping ages (75 and 80 years), and screening intervals (2 and 3 years). We assumed full adherence in all screening scenarios. The baseline scenario was modelled to project the trend in gastric cancer in the absence of a national endoscopic screening policy.

For the cost effectiveness analysis in this study, the model repeatedly simulated 10 million individuals born between 1965 and 1985 in all screening scenarios and followed them from age 20 years until either death or age 100 years. Each comparison between screening scenarios was therefore based on a group of individuals with an identical distribution of birth year, sex, *H. pylori* infection, smoking behaviors, potential time of death from background mortality, and adherence to screening strategies.

**Model calibration**

Model calibration, also referred as model fitting, allows the model outputs to align with observed empirical data. We calibrated the model to reproduce the population-based cancer registries data on age- and sex-specific incidence and stage distribution of gastric cancer from 2006 to 2008 [12]. The population-based cancer registry has de-identified, individual-level data on 1.2 million gastric cancer cases diagnosed from 1994 to 2013. We included anatomic sites C16.1–9. The histologic types include: adenocarcinoma (8140), tubular adenocarcinoma (8211), intestinal type adenocarcinoma (8144), papillary adenocarcinoma (8260/3), and mucinous adenocarcinoma (8480/3).

This study specified calibration targets to identify the best-fit parameter set that produce model outputs matching up to the observed data: age- and sex-specific targets for incidence rate from 2006 to 2008; and stage distribution targets from 2006 to 2008. We defined the initial search bounds for calibration by conducting a literature search (Table S1). To inform the plausible range for the transition probabilities with a single estimate from the literature, we used 0.5 to 2 times multipliers as the initial search bound. We explored parameter space systematically by performing 6000 independent searches with 1 million individuals in each search. Of 6000 resamples, differences between model output and observed data of each parameter set were measured using goodness-of-fit statistics of the sum-of-squared error [38]. The best-fit parameter set was defined as the parameter set with the lowest goodness-of-fit score [38]. We reported the uncertainty intervals using the top 50 best fitted parameter sets, which allowed incorporation of parameter (second-order) uncertainty.

**Fit to calibration targets**

The model calibration showed that the predicted values were 100% fitted to the observed values. Figure S2, Figure S3, Figure S4, and Figure S5 present the model-predicted values and observed values. Our microsimulation model accurately reproduced the age- and sex- specific incidence rates and stage distribution to the observed trends in population-based cancer registries from 2006 to 2008.

**Figure S2. Model predicted and observed gastric cancer incidence, both sexes**

| **A** |
| --- |
| **B** |
| **C** |

Note: Comparisons of the observed and model predicted age-specific NCGA incidence of individuals aged 25 years or above; in (A) 2006, (B) 2007, and (C) 2008. The observed age-specific gastric cancer incidence are indicated by black hollow dots. The dotted lines indicate mean predicted age-specific incidence of gastric cancer, and shaded areas indicate uncertainty intervals. NCGA, noncardia gastric adenocarcinoma.

**Figure S3. Model predicted and observed gastric cancer incidence, women**

| **A** |
| --- |
| **B** |
| **C** |

Note: Comparisons of the observed and model predicted age-specific NCGA incidence of women aged 25 years or above; in (A) 2006, (B) 2007, and (C) 2008. The observed age-specific gastric cancer incidence are indicated by black hollow dots. The dotted lines indicate mean predicted age-specific incidence of gastric cancer, and shaded areas indicate uncertainty intervals. NCGA, noncardia gastric adenocarcinoma.

**Figure S4. Model predicted and observed gastric cancer incidence, men**

| **A** |
| --- |
| **B** |
| **C** |

Note: Comparisons of the observed and model predicted age-specific NCGA incidence of men aged 25 years or above; in (A) 2006, (B) 2007, and (C) 2008. The observed age-specific gastric cancer incidence are indicated by black hollow dots. The dotted lines indicate mean predicted age-specific incidence of gastric cancer, and shaded areas indicate uncertainty intervals. NCGA, noncardia gastric adenocarcinoma.

**Figure S5. Model predicted and observed stage distribution of gastric cancer**

| **A** | **B** | **C** |
| --- | --- | --- |

Note: Comparisons of the observed stage distributions of NCGA and model predicted stage distributions of NCGA for individuals aged 25 years or above; in (A) 2006, (B) 2007, and (C) 2008. The observed stage distributions of NCGA are indicated by blue bars. The red bars indicate mean model prediction on stage distributions of NCGA, and error bars indicate uncertainty intervals. NCGA, noncardia gastric adenocarcinoma.

**Model validation**

To validate the model, we assessed the model predictive ability from the 50 best fit parameter sets on empirical data that were not used in calibration process, namely gastric cancer mortality on vital statistics from 1994 to 2013 [12,39].

**Fit to validation targets**

The external-validation analyses demonstrated coverage estimates of 100% for predicted crude mortality from 1994 to 2013. Validation of the microsimulation model is shown in Figure S6.

**Figure S6. Predicted gastric cancer mortality**

|  |
| --- |

Note: The crude mortality rate from NCGA from vital statistics between 1994 and 2013 and as predicted by the model. The observed crude mortality from the vital statistics from 1994 to 2013 are indicated by dark blue hollow dots. The horizontal lines indicate the range of model predicted crude mortality rate of gastric cancer. NCGA, noncardia gastric adenocarcinoma.

**CHEERS checklist**

**Table S5. CHEERS checklist—Items to include when reporting economic evaluations of health interventions**

| **Section/item** | **Item No.** | **Recommendation** | **Reported on page No./line No.** |
| --- | --- | --- | --- |
| **Title and abstract** |  |  |  |
| Title | 1 | Identify the study as an economic evaluation or use more specific terms such as “cost-effectiveness analysis”, and describe the interventions compared. | page 1 |
| Abstract | 2 | Provide a structured summary of objectives, perspective, setting, methods (including study design and inputs), results (including base case and uncertainty analyses), and conclusions. | page 2 and 3 |
| **Introduction** |  |  |  |
| Background and objectives | 3 | Provide an explicit statement of the broader context for the study. | page 3, line 64 to 75 |
|  |  | Present the study question and its relevance for health policy or practice decisions. | page 4, line 77 to 82 |
| **Methods** |  |  |  |
| Target population and subgroups | 4 | Describe characteristics of the base case population and subgroups analysed, including why they were chosen. | page 4, line 86 to 91;  page 6, line 139 to 141 |
| Setting and location | 5 | State relevant aspects of the system(s) in which the decision(s) need(s) to be made. | page 4, line 86 to 91 |
| Study perspective | 6 | Describe the perspective of the study and relate this to the costs being evaluated. | page 6, line 139 to 141;  Table 1 |
| Comparators | 7 | Describe the interventions or strategies being compared and state why they were chosen. | page 6, line 126 to 129 |
| Time horizon | 8 | State the time horizon(s) over which costs and consequences are being evaluated and say why appropriate. | page 6, line 139 to 141 |
| Discount rate | 9 | Report the choice of discount rate(s) used for costs and outcomes and say why appropriate. | page 6, line 152 to 153 |
| Choice of health outcomes | 10 | Describe what outcomes were used as the measure(s) of benefit in the evaluation and their relevance for the type of analysis performed. | page 6, line 141 to 144 |
| Measurement of effectiveness | 11a | *Single study-based estimates:*Describe fully the design features of the single effectiveness study and why the single study was a sufficient source of clinical effectiveness data. |  |
|  | 11b | *Synthesis-based estimates*: Describe fully the methods used for identification of included studies and synthesis of clinical effectiveness data. | Supplementary,  page 3, 9, and 10 |
| Measurement and valuation of preference based outcomes | 12 | If applicable, describe the population and methods used to elicit preferences for outcomes. | not applicable |
| Estimating resources and costs | 13a | *Single study-based economic evaluation:*Describe approaches used to estimate resource use associated with the alternative interventions. Describe primary or secondary research methods for valuing each resource item in terms of its unit cost. Describe any adjustments made to approximate to opportunity costs. |  |
|  | 13b | *Model-based economic evaluation:*Describe approaches and data sources used to estimate resource use associated with model health states. Describe primary or secondary research methods for valuing each resource item in terms of its unit cost. Describe any adjustments made to approximate to opportunity costs. | page 4, 5, line 85 to 123;  Table 1 |
| Currency, price date, and conversion | 14 | Report the dates of the estimated resource quantities and unit costs. Describe methods for adjusting estimated unit costs to the year of reported costs if necessary. Describe methods for converting costs into a common currency base and the exchange rate. | Table 1;  page 6, 7, line 153 to 154 |
| Choice of model | 15 | Describe and give reasons for the specific type of decision-analytical model used. Providing a figure to show model structure is strongly recommended. | Figure 1 and Figure 2 |
| Assumptions | 16 | Describe all structural or other assumptions underpinning the decision-analytical model. | Supplementary Table S2;  Table 1 |
| Analytical methods | 17 | Describe all analytical methods supporting the evaluation. This could include methods for dealing with skewed, missing, or censored data; extrapolation methods; methods for pooling data; approaches to validate or make adjustments (such as half cycle corrections) to a model; and methods for handling population heterogeneity and uncertainty. | page 4, 5, line 93 to 108;  Supplementary page 12 to 16 |
| **Results** |  |  |  |
| Study parameters | 18 | Report the values, ranges, references, and, if used, probability distributions for all parameters. Report reasons or sources for distributions used to represent uncertainty where appropriate. Providing a table to show the input values is strongly recommended. | Table 1 |
| Incremental costs and outcomes | 19 | For each intervention, report mean values for the main categories of estimated costs and outcomes of interest, as well as mean differences between the comparator groups. If applicable, report incremental cost-effectiveness ratios. | page 8, 9 line 187 to 215  Table 2; Figure 4 |
| Characterising uncertainty | 20a | *Single study-based economic evaluation:*Describe the effects of sampling uncertainty for the estimated incremental cost and incremental effectiveness parameters, together with the impact of methodological assumptions (such as discount rate, study perspective). |  |
|  | 20b | *Model-based economic evaluation:*Describe the effects on the results of uncertainty for all input parameters, and uncertainty related to the structure of the model and assumptions. | page 9, 10 line 218 to 232; Figure 5 |
| Characterising heterogeneity | 21 | If applicable, report differences in costs, outcomes, or cost-effectiveness that can be explained by variations between subgroups of patients with different baseline characteristics or other observed variability in effects that are not reducible by more information. | not applicable |
| **Discussion** |  |  |  |
| Study findings, limitations, generalisability, and current knowledge | 22 | Summarise key study findings and describe how they support the conclusions reached. Discuss limitations and the generalisability of the findings and how the findings fit with current knowledge. | page 10, 11, 12, 13  line 235 to 307; |
| **Other** |  |  |  |
| Source of funding | 23 | Describe how the study was funded and the role of the funder in the identification, design, conduct, and reporting of the analysis. Describe other non-monetary sources of support. | Page 13, 14 |
| Conflicts of interest | 24 | Describe any potential for conflict of interest of study contributors in accordance with journal policy. In the absence of a journal policy, we recommend authors comply with International Committee of Medical Journal Editors recommendations. | Page 13 |

**Trends in gastric cancer incidence rate**

**Figure S7. Trends in gastric cancer incidence rates by age (ages 40–49 and ages 50–59) and sex, 1993 to 2014**

|  |
| --- |

Note: Data source: high-quality population-based cancer registries (Yamagata, Fukui, and Nagasaki).
